# European Network of Rare Disease Help Lines (ENRDHLs) – caller profile analysis 2013

**DOI:** 10.1186/1750-1172-9-S1-O13

**Published:** 2014-11-11

**Authors:** Georgi Iskrov, François Houÿez

**Affiliations:** 1 Institute for Rare Diseases, 4000 Plovdiv, Bulgaria; 2 Department of Social Medicine and Public Health, Faculty of Public Health, Medical University of Plovdiv, 4000 Plovdiv, Bulgaria; 3 European Organisation for Rare Diseases (EURORDIS), 75014 Paris, France

## Background

Generation and dissemination of information has been a crucial area for actions in the field of rare diseases. In this context, rare disease patients and their experience are recognised as a unique source of information that could trigger important developments for all stakeholders.

The European Network of Rare Disease Help Lines (ENRDHLs) is an initiative led by EURORDIS. ENRDHLs was created within the EU-funded Rapsody with the main aim of improving the quality of services provided by European help lines for rare diseases. In order to fulfill this objective, various rare diseases information services from across Europe came together to share expertise and propose ways in which the network could support other services in Europe.

## Materials and methods

The Caller Profile Analysis is one of the mandatory commitments for the ENRDHLs members. It is a cross-sectional survey that describes the rare disease helpline services, provided by the participating partners for a specific month. The 2013 survey covered all enquiries received from 1 to 30 October 2013.

## Results

12 rare disease helplines from 8 countries (France, Italy, Spain, Switzerland, Portugal, Romania, Bulgaria and Croatia) took part in the Caller Profile Analysis 2013, giving information on 1 672 enquiries. The typical user would be a female patient, aged 30-50 years old. The overall volume of enquiries served has been relatively stable for the period of 2011-2013, with an increased number of enquiries on centres of expertise and a decreased number of enquires on information on specific rare diseases. These combined outcomes demonstrate the still unmet needs of rare disease patients for information. Patients hope that this gap could be efficiently filled by the ongoing designation of centres of expertise throughout Europe.

## Conclusions

Despite the acknowledged progress in rare disease field at both EU and national level and the increased role of social media, traditional helpline information services do have their importance for rare diseases stakeholders and especially for patients. They have a proven record for effectively empowering patients, giving them the capacity to communicate, to collaborate and to advocate for rare disease policy and actions. And empowered patients are the common denominator that sticks together all rare disease stakeholders and make changes occur.

